# Reductions in intestinal Clostridiales precede the development of nosocomial *Clostridium difficile* infection

**DOI:** 10.1186/2049-2618-1-18

**Published:** 2013-06-28

**Authors:** Caroline Vincent, David A Stephens, Vivian G Loo, Thaddeus J Edens, Marcel A Behr, Ken Dewar, Amee R Manges

**Affiliations:** 1 Department of Microbiology and Immunology, McGill University, 3775 University Street, Montréal, Québec H3A 2B4, Canada; 2 McGill University and Génome Québec Innovation Centre, 740 Dr. Penfield Avenue, Montréal, Québec H3A 0G1, Canada; 3 Department of Mathematics and Statistics, McGill University, 805 Sherbrooke Street West, Montréal, Québec H3A 0B9, Canada; 4 The Research Institute of the McGill University Health Centre, 2155 Guy Street, Montréal, Québec H3H 2R9, Canada; 5 Devil’s Staircase Consulting, 693 Osborne Road East, North Vancouver, British Columbia V7N 1M8, Canada; 6 Department of Human Genetics, McGill University, 1205 Dr. Penfield Avenue, Montréal, Québec H3A 1B1, Canada; 7 School of Population and Public Health, University of British Columbia, 2206 East Mall, Vancouver, British Columbia V6T 1Z3, Canada

**Keywords:** Intestinal microbiota, *Clostridium difficile* infection, 16S rRNA gene sequencing, Clostridiales Incertae Sedis XI

## Abstract

**Background:**

Antimicrobial use is thought to suppress the intestinal microbiota, thereby impairing colonization resistance and allowing *Clostridium difficile* to infect the gut. Additional risk factors such as proton-pump inhibitors may also alter the intestinal microbiota and predispose patients to *Clostridium difficile* infection (CDI). This comparative metagenomic study investigates the relationship between epidemiologic exposures, intestinal bacterial populations and subsequent development of CDI in hospitalized patients. We performed a nested case–control study including 25 CDI cases and 25 matched controls. Fecal specimens collected prior to disease onset were evaluated by 16S rRNA gene amplification and pyrosequencing to determine the composition of the intestinal microbiota during the at-risk period.

**Results:**

The diversity of the intestinal microbiota was significantly reduced prior to an episode of CDI. Sequences corresponding to the phylum Bacteroidetes and to the families Bacteroidaceae and Clostridiales Incertae Sedis XI were depleted in CDI patients compared to controls, whereas sequences corresponding to the family Enterococcaceae were enriched. In multivariable analyses, cephalosporin and fluoroquinolone use, as well as a decrease in the abundance of Clostridiales Incertae Sedis XI were significantly and independently associated with CDI development.

**Conclusions:**

This study shows that a reduction in the abundance of a specific bacterial family - Clostridiales Incertae Sedis XI - is associated with risk of nosocomial CDI and may represent a target for novel strategies to prevent this life-threatening infection.

## Background

*Clostridium difficile* infection (CDI) is the leading cause of nosocomial diarrhea. The incidence and severity of CDI have been rising over the last decade and outbreaks continue to occur across the globe [1]. The changing epidemiology has been linked in part to the emergence of hypervirulent strains of *C. difficile* that are resistant to fluoroquinolones [2]. During the major North American outbreak of 2003 to 2005, the proportion of complicated CDI cases requiring colectomy rose to 18% and fatality rates reached 25% [3,4]. Recognized risk factors for CDI include advanced age, severe underlying illness, previous hospitalization, prolonged hospital stay, and most importantly, exposure to antimicrobials [5]. Broad-spectrum antimicrobial agents are presumed to disrupt the indigenous intestinal microbiota, thereby impairing colonization resistance and allowing the establishment and proliferation of *C. difficile* in the gut. Although nearly all classes of antimicrobial agents have been associated with CDI, clindamycin, penicillins, cephalosporins, and more recently fluoroquinolones seem to pose the greatest risk [5-7].

Other medications besides antimicrobials may also alter the intestinal microbiota and predispose patients to CDI. Gastric-acid suppressive agents like proton-pump inhibitors (PPIs), may act synergistically with antimicrobial agents to disrupt the intestinal microbiota and contribute to CDI development [8]. Epidemiologic evidence has demonstrated an increased risk of nosocomial CDI in patients receiving PPI therapy, often concurrently with antimicrobial agents [9,10]. *C. difficile*-induced inflammation is another factor that may, in conjunction with antimicrobial use, affect the integrity of the intestinal microbiota. Research based on mouse colitis models suggests that intestinal inflammation elicited during colonization by enteric pathogens such as *Salmonella* and *C. difficile* suppresses the indigenous microbiota, allowing these invaders to grow unimpeded [11].

The objective of this study was to examine the complex relationships between epidemiologic exposures, intestinal bacterial populations, and subsequent development of CDI in hospitalized patients. In a previous investigation, we used a microarray with a limited set of 16S rRNA probes to contrast the composition of the fecal microbiota between patients who later developed CDI (cases) and hospitalized controls. In this earlier study, Firmicutes and Bacteroidetes were found to be significantly and independently associated with CDI development [12]. In order to validate and expand these initial results, we re-assessed these valuable pre-disease fecal samples by implementing gold-standard 16S rRNA gene sequencing to obtain a comprehensive survey of the bacterial taxa that are present in the intestinal tract of patients, and by employing statistical approaches to appropriately deal with patients’ complex exposure histories and the high-dimensional nature of the sequencing data. As the composition of the intestinal microbiota is the unifying theme of this study, we also adjusted our target epidemiologic exposure window to focus only on medications received prior to stool collection in each patient. We specifically examined (i) profiles of intestinal microbiota diversity across patients, (ii) differences in the pre-disease composition of the intestinal microbiota between CDI cases and control patients, (iii) the association between intestinal bacterial populations and risk of CDI after adjusting for exposure to epidemiologic factors, and (iv) the relationship between epidemiologic exposures and intestinal microbiota composition. We report that distinctive features of the intestinal microbiota are associated with CDI risk in hospitalized patients.

## Methods

### Study design and subjects

Between September 2006 and May 2007, a total of 599 hospitalized patients were enrolled in a prospective cohort study at the Royal Victoria Hospital, Montréal. A detailed description of the cohort study is available in Loo *et al*. [13]. During the study period, 31 patients experienced one or more defined episodes of CDI. Fecal specimens collected before the onset of the first CDI episode (if multiple occurred) were available for 25 of these patients (cases), and 25 matched controls were selected for inclusion in a nested case–control study. Case patients were matched to controls based on sex, age (± 5 years) and date of hospitalization (± 2 months). For this study, a single rectal swab was obtained from each study subject within 7 days of admission to the hospital. A questionnaire was administered to all study patients and collected information concerning demographics, reason for admission, date of admission and discharge, previous hospitalization, underlying disease severity (based on Charlson index), CDI diagnosis, and use of various medications in the 8 weeks prior to hospital admission and during hospitalization. Detailed medication exposures for all patients included in the study are provided in Additional file 1: Table S1. Information on nasogastric intubation, aminoglycoside and metronidazole use was collected by the larger study, but these variables were excluded from further analyses as exposure was unknown for a large number of patients. We included exposure to intravenous vancomycin in our analyses, as evidence suggests that substantial amounts of the drug can be excreted in the bowel and may therefore affect the intestinal microbiota [14]. All participants provided informed written consent. The human subjects’ protocols for the cohort and case–control studies were approved by the Royal Victoria Hospital Internal Review Board as well as the McGill University Institutional Review Board (BMB 05–014).

### Definitions

CDI was defined by the larger cohort study [13] as follows: (i) the presence of diarrhea and a positive *C. difficile* cytotoxin assay or toxigenic culture, (ii) the presence of diarrhea without an alternate explanation and an endoscopic diagnosis of pseudomembranes, or (iii) a pathological diagnosis of CDI. Diarrhea was defined as three loose stools within 24 hours for one or more days. Toxigenic *C. difficile* culture was performed according to standard procedures [13].

### Fecal specimen processing

Fecal DNA was isolated with use of the DNA IQ System (Promega Corporation, Madison, WI, USA) and subjected to whole-genome amplification using the illustra GenomiPhi V2 DNA Amplification Kit (GE Healthcare Bio-Sciences Corporation, Piscataway, NJ, USA). Whole-genome amplification was necessary because of limited fecal material available from study subjects and to ensure sufficient DNA quantities for subsequent steps. The GenomiPhi kit was previously shown to generate the least amount of bias compared to other DNA amplification methods [15]. The amplified DNA was purified with the PureLink PCR Purification Kit (Life Technologies Inc., Burlington, ON, Canada).

### 16S rRNA gene amplification and sequencing

16S rRNA gene amplification was performed as described in the Human Microbiome Project Provisional 16S 454 Protocol [16]. Pyrosequencing was performed at the McGill University and Génome Québec Innovation Centre using Roche/454 GS-FLX Titanium technology.

### Bioinformatic analysis

The open-source software mothur [17] was used to process sequences from 16S rRNA gene libraries. Sequences corresponding to V1-V3 and V3-V5 were binned according to primer sequence and analyzed separately. Reads containing ambiguous bases (Ns), homopolymer runs greater than 8 bases, inexact match to the MID tag, or more than two differences from the primer sequence were excluded from the dataset. Remaining sequences were aligned against mothur’s Silva reference database using the NAST algorithm [18]. Potential chimeric sequences were detected with mothur’s implementation of the ChimeraSlayer tool [19] and removed accordingly. Rare sequence variants that likely arose from pyrosequencing errors were merged with their more abundant parent sequence using a single-linkage pre-clustering algorithm [17].

To determine the proportion of sequences corresponding to *C. difficile* in patient samples, the entire set of high-quality reads from V1-V3 and V3-V5 were BLASTed against a database of 42 annotated 16S rRNA genes representing 4 finished *C. difficile* genomes. BLAST hits with ≥99% identity and ≥99% coverage were considered to be *C. difficile*.

In all other analyses, we controlled for differences in sequencing depth by normalizing the number of high-quality reads obtained for each sample. For taxonomic analyses, sequences were annotated using phylum and family-level assignments with the Bayesian classifier implemented by the Ribosomal Database Project [20]. A minimum confidence threshold of 80% was required for each assignment. For diversity analyses, sequences were grouped into species-level operational taxonomic units (OTUs) using the average neighbor clustering algorithm [21]. OTUs were defined as groups of 16S sequences sharing at least 97% pairwise identity. The heatmap with hierarchical clustering was generated with R [22]. Principal coordinate analysis (PCoA) was performed with mothur [17].

### Statistical analysis

Normalized sequence counts by bacterial phylum and family were log-transformed, in order to minimize undue influence from extreme values. In statistical models including epidemiologic variables, we considered those exposures that occurred in the 8 weeks before, as well as during hospitalization, until the date of stool collection. In univariate analyses, we first used a penalized least-squares regression approach (LASSO) to select important predictor variables [23]. The association between intestinal bacterial taxa and CDI development was evaluated by logistic regression. The association between epidemiologic exposures and intestinal microbiota composition was assessed by Poisson regression. An interaction term was included in the latter model to account for the effect of disease status on intestinal bacterial populations. Multivariable logistic regression was used to identify which bacterial taxa remained independently associated with CDI development after adjusting for the effects of epidemiologic exposures. Selected variables included medications that were administered to at least 8 out of 50 patients, as well as bacterial taxa that were associated with CDI in the univariate analysis. Multiscale bootstrapping and analysis of molecular variance (AMOVA) tests were performed with pvclust and mothur, respectively [17,24]. All other statistical analyses were performed with Stata (version 11, StataCorp) or R [22].

## Results

### Subject characteristics

Twenty-five patients with CDI and 25 age- and sex-matched controls were retrospectively sampled from subjects enrolled in a large cohort study at the Royal Victoria Hospital, Montréal. Characteristics of the study patients are reported in Table 1. Case and control patients had similar risk of exposure to *C. difficile*; the duration of hospitalization (based on the time from admission until CDI diagnosis for case patients or until discharge for control patients) was not significantly different between the groups (*P* = 0.26, by Mann–Whitney *U*-test). Among CDI cases, three patients experienced more than one defined episode of CDI during the study period. A single fecal specimen was obtained from each patient shortly after hospital admission, but before the onset of the first CDI episode (if multiple occurred) in the cases (during the at-risk period). Among case patients, the median interval of time between stool collection and CDI diagnosis was 5 days.

**Table 1:** Characteristics of study patients

**Variable**	**CDI cases (n = 25)**	**Controls (n = 25)**
Age, mean years ± SD	70 ± 12.8	69 ± 12.5
Male sex	12 (48)	12 (48)
Charlson comorbidity index, median score (IQR)	1 (1–3)	2 (1–3)
Duration of hospitalization^a^, median days (IQR)	7 (4–28)	11 (8–17)
Hospitalization in past 12 months	19 (76)	15 (60)
Reason for hospital admission		
Cardiac problem	9 (36)	10 (40)
Gastrointestinal problem	10 (40)	6 (24)
Pulmonary problem	2 (8)	3 (12)
Renal disease	2 (8)	1 (4)
Other^b^	2 (8)	5 (20)
Medication use^c^		
H2 blocker	7 (29)	4 (16)
Nonsteroidal anti-inflammatory drug	15 (65)	12 (48)
Proton-pump inhibitor	11 (46)	14 (56)
Steroid	3 (13)	1 (4)
Any antimicrobial agent	21 (91)	13 (52)
Cephalosporin^d^	10 (42)	5 (20)
Fluoroquinolone^e^	9 (38)	4 (16)
Macrolide	0 (0)	1 (4)
Penicillin	0 (0)	1 (4)
Penicillin with β-lactamase inhibitor	7 (30)	3 (12)
Vancomycin^f^	5 (22)	3 (12)

Data are number (%) of subjects unless otherwise specified. *CDIClostridium difficile* infection, *SD* standard deviation, *IQR* interquartile range. ^a^Duration until CDI diagnosis for case patients or duration until discharge for control subjects. ^b^Other reasons include acquired immunodeficiency syndrome, cancer, breast surgery, anemia, osteomyelitis, neurological or rheumatological problems. ^c^Defined as use within 8 weeks before or during hospitalization, until stool collection. Information on steroid, nonsteroidal anti-inflammatory drug, any antimicrobial agent, penicillin with β-lactamase inhibitor and vancomycin use was available for 23 out of 25 case patients; information on other medications was available for 24 out of 25 case patients. None of the patients were exposed to probiotics, carbapenem, clindamycin, gatifloxacin, levofloxacin, linezolid, tetracycline or trimethoprim-sulfamethoxazole prior to stool collection. ^d^Includes exposure to first-, second-, and third-generation cephalosporins. ^e^Includes exposure to ciprofloxacin and moxifloxacin. ^f^Intravenous administration.

### 16S rRNA gene sequencing

Fecal specimens (n = 50; one per subject) were evaluated by 16S rRNA gene amplification and pyrosequencing to determine the composition of the intestinal microbiota. A total of 4.1 × 10^5^ high-quality reads (range 2,676-31,641 per subject) from two segments (V1-V3 and V3-V5) of the 16S rRNA gene were analyzed. The taxonomic profiles generated from the amplification of V1-V3 and V3-V5 were in accordance, with a median Pearson correlation coefficient of 0.90. The majority of sampled sequences corresponded to Firmicutes (51% and 55%), Bacteroidetes (34% and 30%) and Proteobacteria (8% and 11%) (percentage of V1-V3 and V3-V5 sequence sets, respectively).

Based on toxigenic *C. difficile* culture assays performed by the larger cohort study [13], six patients were found to be culture-positive on the same date as the fecal specimen used for sequencing was collected. Of these, four patients went on to develop CDI (cases), and two were asymptomatically colonized (controls). In five of these six culture-positive patients, we detected sequences corresponding to *C. difficile*; sequences could not be detected in one of the two asymptomatically colonized controls. There were two instances where *C. difficile* V1-V3 or V3-V5 sequences comprised >1% of the sequence data, and both of these patients showed clinical manifestation of CDI within the subsequent two days.

### Intestinal microbiota diversity

**Figure 1:**
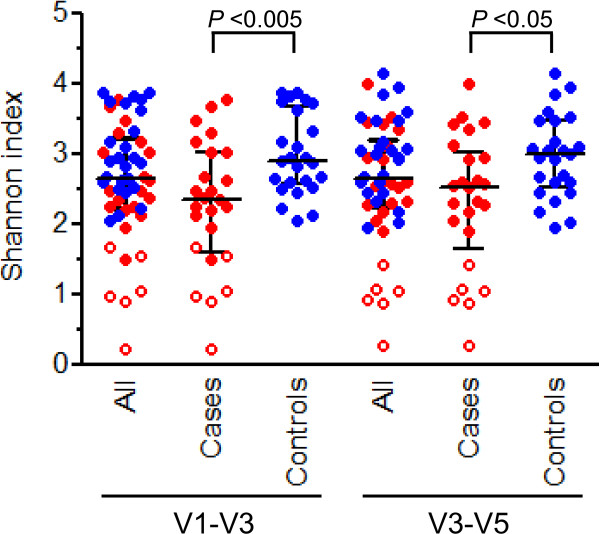
**Diversity of the intestinal microbiota across *Clostridium difficile *infection (CDI) cases and control subjects.** The 16S rRNA gene sequences were clustered into operational taxonomic units (OTUs) defined by ≥97% nucleotide sequence identity. Case patients (n = 25) are colored in red and control patients (n = 25) are colored in blue. Patients with the lowest degree of intestinal biodiversity (n = 6; all of these patients are cases) are shown with open circles. Results are presented for both V1-V3 and V3-V5 sequence sets. Horizontal lines represent the median and interquartile range. *P* values were determined by Mann–Whitney *U*-test.

**Figure 2:**
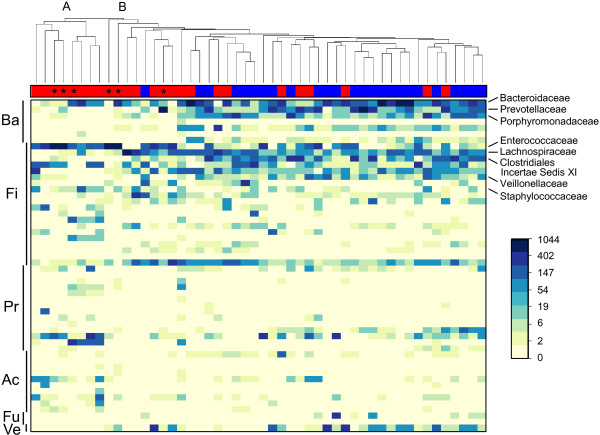
**Intestinal microbiota profiles across *Clostridium difficile *infection (CDI) cases and control subjects.** The heatmap shows the abundance of V1-V3 sequences by bacterial family (rows) across all patients (columns). Sequence counts were normalized in order to obtain an equivalent number of reads for each sample. The dendrogram shows hierarchical clustering (unweighted pair group method with arithmetic mean) of microbial communities using Canberra distance metric. The bar on the top indicates disease status for each patient: cases (n = 25) are in red and controls (n = 25) are in blue. Patients with the lowest degree of intestinal biodiversity (n = 6; all of these patients are cases) are marked with an asterisk. The staggered bars on the left indicate phylum affiliations: Ba, Bacteroidetes; Fi, Firmicutes; Pr, Proteobacteria; Ac, Actinobacteria; Ve, Verrucomicrobia; Fu, Fusobacteria. Other phyla (Lentisphaerae, Spirochaetes, Synergistetes, Tenericutes, Cyanobacteria and TM7) and reads that were unclassified at the phylum level, which altogether represent ≤6.3% of the reads/patient, are not depicted. Relevant bacterial families are listed on the right of the figure. The color gradient is proportional to the logarithm of sequence abundance from 0 to 1,044 reads, as indicated by the scale.

After normalizing for read counts across samples we observed variability in intestinal biodiversity across patients. Patient samples exhibited a Shannon Index value range of 0.2 to 3.9 according to V1-V3 data and a range of 0.3 to 4.2 according to V3-V5 data (Figure 1). Reduced biodiversity was significantly related to incipient CDI. Based on V1-V3 data, all 8 patients (7 patients based on V3-V5 data) with the lowest degree of microbial diversity developed CDI, as did 16 (14 based on V3-V5 data) of the 20 patients with the least diverse microbiota (Figure 1). Sequence abundances by bacterial family across patients are presented as a heatmap with hierarchical clustering in Figure 2. There was substantial inter-individual variation in the composition of the fecal microbiota. Patient samples were divided in two main profile clusters, A and B, with 93% and 89% support, respectively, by multiscale bootstrapping with 100,000 replicates. Cluster A contained a subset of 8 CDI cases, while cluster B contained all 25 controls unevenly mixed with 17 cases.

**Figure 3:**
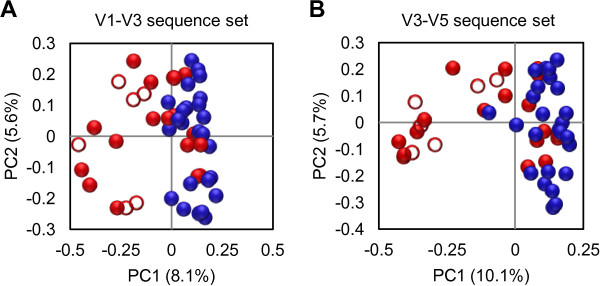
**Intestinal community clustering of *Clostridium difficile *infection (CDI) cases and control subjects based on principal coordinate analysis (PCoA).** Results are presented for **(A)** V1-V3 and **(B)** V3-V5 sequence sets. Case patients (n = 25) are colored in red and control patients (n = 25) are colored in blue. Patients with the lowest degree of intestinal biodiversity (n = 6; all of these patients are cases) are shown with open circles. The percentage of variation explained by each principal coordinate (PC) is indicated on the corresponding axis.

Differences in intestinal community membership across patients were assessed by PCoA of Jaccard distances (Figure 3). In both V1-V3 and V3-V5 sequence sets, the large majority of intestinal samples from cases and controls clustered separately in the PCoA plot (*P* <0.001 for both V1-V3 and V3-V5 sequence sets, by AMOVA), although the overall magnitude of variation explained by principal coordinate 1 was modest (8.1% for V1-V3 and 10.1% for V3-V5). Samples from case patients also displayed a greater level of heterogeneity in their community membership, as shown by the larger within-group distances in cases compared to controls (Figure 3; *P* <0.0001 for both V1-V3 and V3-V5 sequence sets, by Mann–Whitney *U*-test).

### Association between intestinal microbiota composition and *Clostridium difficile* infection (CDI)

**Figure 4:**
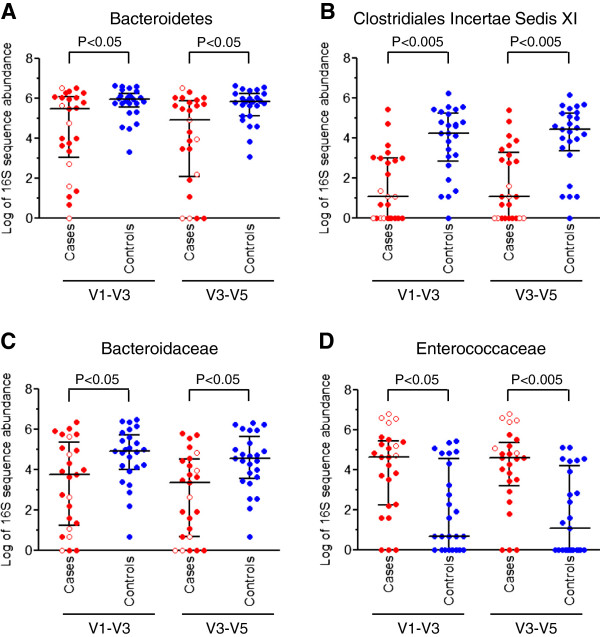
**Intestinal bacterial taxa exhibiting significant differences in abundance between *Clostridium difficile *infection (CDI) cases and control subjects.** The scatter plots show log-transformed 16S sequence counts for the corresponding bacterial **(A)** phylum or **(B-D)** family in cases (n = 25) versus controls (n = 25). Results are presented for both V1-V3 and V3-V5 sequence sets. Patients with the lowest degree of intestinal biodiversity (n = 6; all of these patients are cases) are shown with open circles. Horizontal lines represent the median and interquartile range. *P* values were determined by logistic regression.

We examined the relationship between intestinal bacterial taxa and subsequent development of CDI. At the phylum level, the abundance of Bacteroidetes was significantly lower in cases compared to controls (Figure 4A). At the family level, sequences corresponding to Clostridiales Incertae Sedis XI (Figure 4B) and Bacteroidaceae (Figure 4C) were depleted in patients with CDI, while sequences assigned to Enterococacceae were enriched (Figure 4D).

### Association between intestinal microbiota composition and *Clostridium difficile* infection (CDI) after adjustment for epidemiologic exposures

We used a multivariable analysis to control for the influence of antimicrobials and other medications on the distribution of bacterial taxa that are related to CDI development (Table 2). At the phylum level, cephalosporin and fluoroquinolone use were significantly associated with CDI development, while a decrease in the proportion of Bacteroidetes was of borderline significance. At the family level, cephalosporin exposure and a reduction in the frequency of Clostridiales Incertae Sedis XI were significant risk factors for CDI, while fluoroquinolone exposure was of borderline significance.

**Table 2:** **Multivariable analysis of epidemiologic exposures and intestinal bacterial taxa related to** ***Clostridium difficile*** **infection (CDI) development**

**Variable**	**Phylum-level analysis**			
	**V1-V3 sequence set**		**V3-V5 sequence set**	
	***P*** **value**^**a**^	**Coefficient sign**^**b**^	***P*** **value**^**a**^	**Coefficient sign**^**b**^
Bacterial phylum				
Bacteroidetes	0.048	-	0.061	-
Shannon diversity	0.187	-	0.455	-
Medication use^c^				
H2 blocker	0.319	-	0.271	-
Nonsteroidal anti-inflammatory drug	0.684	+	0.989	+
Proton-pump inhibitor	0.443	-	0.467	-
Cephalosporin	0.016	+	0.009	+
Fluoroquinolone	0.038	+	0.018	+
Penicillin with β-lactamase inhibitor	0.228	+	0.424	+
Vancomycin^d^	0.278	+	0.116	+
	**Family-level analysis**			
	**V1-V3 sequence set**		**V3-V5 sequence set**	
	***P *** **value**^**a**^	**Coefficient sign**^**b**^	***P *** **value**^**a**^	**Coefficient sign**^**b**^
Bacterial family				
Bacteroidaceae	0.073	-	0.051	-
Clostridiales Incertae Sedis XI	0.015	-	0.025	-
Enterococcaceae	0.942	+	0.246	+
Shannon diversity	0.728	+	0.238	+
Medication use^c^				
H2 blocker	0.384	-	0.318	-
Nonsteroidal anti-inflammatory drug	0.921	+	0.605	+
Proton-pump inhibitor	0.674	-	0.558	-
Cephalosporin	0.020	+	0.027	+
Fluoroquinolone	0.045	+	0.061	+
Penicillin with β-lactamase inhibitor	0.692	+	0.850	-
Vancomycin^d^	0.423	+	0.193	+

^a^*P* values were determined by multivariate logistic regression. ^b^A positive sign indicates that 16S sequence abundance (for bacterial taxa) or number of patients exposed (for medications) was higher among patients with CDI than among controls, while a negative sign indicates that 16S sequence abundance or number of patients exposed was lower among patients with CDI. ^c^Defined as use within 8 weeks before or during hospitalization, until stool collection. ^d^Intravenous administration.

### Association between epidemiologic exposures and intestinal microbiota composition

**Figure 5:**
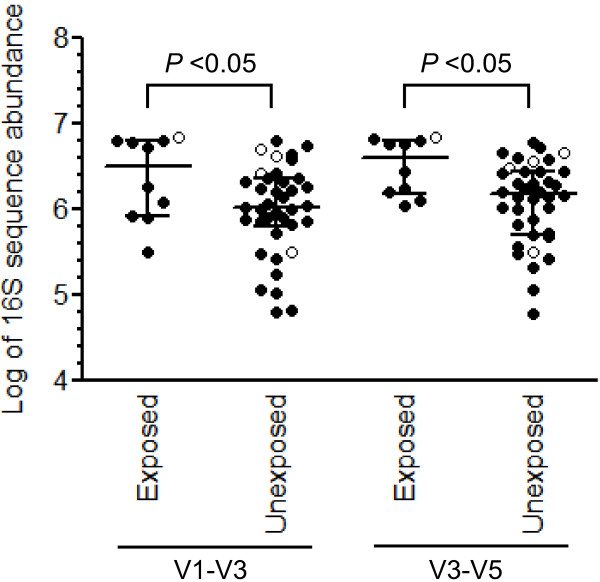
**Exposure to penicillin with β-lactamase inhibitor is associated with an increase in Firmicutes.** The scatter plot shows log-transformed 16S sequence counts for Firmicutes in patients that were exposed (n = 10) or unexposed (n = 38) to penicillin with β-lactamase inhibitor. Results are presented for both V1-V3 and V3-V5 sequence sets. Patients with the lowest degree of intestinal biodiversity (n = 5) are shown with open circles. Horizontal lines represent the median and interquartile range. *P* values were determined by Poisson regression. Note that data on exposure to penicillin with β-lactamase inhibitor was missing in two patients, including one of the patients with the lowest degree of intestinal biodiversity; these are not depicted.

The impact of antimicrobials and other medications on the composition of the intestinal microbiota in patients was examined. We detected a significant association between exposure to penicillin with β-lactamase inhibitor and an increase in the abundance of Firmicutes (Figure 5). No other medications were observed to be associated with differences in intestinal microbiota composition.

### Assessment of six patients with least diverse intestinal microbiota

Six case patients exhibited the lowest degree of microbial diversity (V1-V3 and V3-V5 Shannon Index value <1.7) of all study subjects (Figure 1). Although medication information was missing for one case, there was no indication that the other five low diversity cases differed from the rest of patients in terms of exposure to antimicrobials or other medications (see Additional file 1: Table S1; *P* >0.05 for all medications, by Fisher’s exact test). In the heatmap, these six low diversity cases were spread across clusters A and B and did not share a common taxonomic profile (Figure 2). However, these patients were clearly positioned away from the cluster of controls in the PCoA plot (Figure 3). The enrichment in Enterococacceae was observed in all of the six patients with reduced biodiversity (Figure 4D).

## Discussion

Exposure to antimicrobials or antimicrobials in conjunction with other medications is thought to alter the intestinal microbiota and impair colonization resistance to *C. difficile*. By obtaining fecal specimens in the at-risk period prior to CDI onset, we were able to evaluate the impact of epidemiologic exposures and intestinal microbiota composition on CDI risk. Not only do our results confirm the existence of a compromised gut microbiota in CDI patients, but we were able to identify specific epidemiologic and microbiota factors that are significantly and independently associated with CDI development.

Several studies have observed a reduced microbial diversity in patients with CDI or other diseases, including irritable bowel syndrome and obesity [25-28]. Our results confirm that low diversity is related to CDI development. However, this feature was not found to be an independent predictor of CDI in the multivariable analysis. Reduced diversity may be a non-specific marker of disease.

The levels of Bacteroidetes and Enterococcaceae were markedly altered in patients that were about to experience CDI; however, after adjustment for medication use, these associations were no longer significant. Ferreira *et al*. have suggested that Bacteroidetes may confer resistance to infectious colitis by protecting against pathogen-mediated intestinal inflammation [29]. Intriguingly, the observed increase in Enterococcaceae appeared to be mostly driven by a subset of six cases with the lowest degree of intestinal diversity. Enterococci are opportunistic microorganisms that can, like *C. difficile*, exploit the reduced biodiversity of the intestinal ecosystem to expand their population. This idea is consistent with studies showing increased levels of enterococci in the gut following treatment with extended-spectrum antimicrobial agents [30,31]. In a study by Lawley and colleagues, antibiotic treatment of mice asymptomatically colonized with *C. difficile* resulted in a dramatic reduction in intestinal microbial diversity accompanied by an expansion of *Escherichia coli* and enterococci which triggered the overgrowth of *C. difficile*[32].

In the multivariable analysis, cephalosporin and fluoroquinolone exposure, as well as a decrease in the abundance of Clostridiales Incertae Sedis XI were significantly associated with CDI development. According to current taxonomic lineages, *C. difficile* (which is part of the Peptostreptococaceae family) and the bacterial family Clostridiales Incertae Sedis XI belong to the same order (Clostridiales) [20,33]. Therefore, the depletion of Clostridiales Incertae Sedis XI that preceded CDI onset may indicate an absence of competitive exclusion or other colonization resistance mechanisms operating in the intestinal microbiota of these patients. Studies involving animal models suggest that competition for similar nutrient sources or ecological niches mediated by closely related bacterial groups that are already established in the gut may prevent invasion by pathogenic relatives such as *C. difficile*[34]. A randomized clinical trial to evaluate the safety and efficacy of colonization with non-toxigenic *C. difficile* for the prevention of recurrent CDI is currently underway [35].

We have demonstrated an association between the use of penicillin with β-lactamase inhibitor and an increase in the abundance of Firmicutes. Culture-based analyses of the human fecal microbiota have previously shown that administration of amoxicillin-clavulanic acid (a penicillin with β-lactamase inhibitor) increases the number of aerobic Gram-positive cocci, most of which belong to Firmicutes [36].

In our previous 16S rRNA microarray-based investigation of the same set of samples, we could not establish that low intestinal microbial diversity is associated with CDI development [12]. In this study, high-resolution sequencing along with analyses performed at a lower phylogenetic level allowed us to capture most of the bacterial diversity, and we were able to confirm that reduced diversity is related to CDI. This study also validates our previous observation that an enrichment of Enterococcaceae and a depletion of Bacteroidetes or Clostridiales Incertae Sedis XI are significantly associated with CDI development [12]. We did observe higher levels of Firmicutes among our CDI patients, as reported previously, but the association was not statistically significant in the current sequence-based study (*P* = 0.09, by logistic regression).

Other authors have assessed intestinal microbiota alterations in patients with an initial or recurrent episode of CDI [25-27,37]. However, these investigations did not account for the influence of antimicrobials and other medications in the analysis of microbial profiles associated with CDI. Moreover, previous studies have typically assessed microbiota composition at the time of CDI diagnosis, when the results are likely confounded by the effects of the disease itself (that is, diarrhea and intestinal inflammation) and the effects of CDI treatment on the intestinal ecology. We did observe reduced levels of the Bacteroides-Porphyromonas-Prevotella group and increased levels of facultative anaerobes in patients with CDI, as reported elsewhere, but we did not find a significant association with members of the enterobacteria, bifidobacteria or lactobacilli [25,26,37]. Among our 25 case patients, 3 experienced multiple CDI episodes. We did not observe specific microbiota alterations that could distinguish these patients from other CDI cases and the small number of patients precluded any further analyses. Whether specific microbiota signatures can predict the eventual development of recurrent CDI (as opposed to a single CDI episode) remains to be addressed.

De La Cochetière *et al*. investigated the relationship between dominant gut bacterial species and subsequent acquisition of *C. difficile* in outpatients receiving antimicrobial therapy. Fecal samples obtained prior to the initiation of antimicrobial treatment were analyzed by temporal temperature gradient gel electrophoresis and the resulting microbial profiles could accurately predict the risk of *C. difficile* acquisition in these subjects [38]. Similarly, our results support the idea that certain patients have an existing predisposition to CDI when they are admitted to the hospital; their intestinal microbiota may be less resilient to the effects of antibiotics or more permissive to the invasion of *C. difficile*.

This study is limited by biases inherent to bacterial DNA extraction (due to differential lysis efficiency), whole-genome amplification and 16S rRNA gene amplification (due to species coverage of the primers and variable numbers of 16S rRNA gene copies per genome), which may contribute to under- or over-representation of certain bacterial taxa. Our limited sample size also made it difficult to account for variability in microbiota profiles due to differences in underlying disease and treatment histories across patients. Despite these limitations, important differences in the abundance of key bacterial taxa were apparent and clearly distinguished CDI cases from control patients.

## Conclusions

Although the association between antimicrobial use and CDI is well established, specific alterations to the intestinal microbiota and how they contribute to disease development are poorly described. In this study, we identified specific epidemiologic and microbiota factors that are associated with CDI risk in hospitalized patients. Based on multivariable analyses, independent risk factors for CDI included cephalosporin and fluoroquinolone exposure, as well as a depletion of Clostridiales Incertae Sedis XI. This important novel finding may eventually lead to the elaboration of targeted microbiota interventions to prevent the development of CDI in high-risk patients.

## Abbreviations

CDI: *Clostridium difficile* infection; OTU: Operational taxonomic unit; PCoA: Principal coordinate analysis; PPI: Proton-pump inhibitor; PCR: Polymerase chain reaction; V1-V3: Variable regions 1 to 3 of the 16S ribosomal RNA gene; V3-V5: Variable regions 3 to 5 of the 16S ribosomal RNA gene.

## Competing interests

The authors declare that they have no competing interests.

## Authors’ contributions

CV carried out the study, participated in data analysis and interpretation, and drafted the manuscript. DAS assisted with statistical analyses. VGL generously provided patient epidemiologic information and clinical samples. TJE performed the statistical analyses. MAB critically reviewed the manuscript. KD participated in data analysis and interpretation, and helped to draft the manuscript. ARM conceived and designed the study, participated in data analysis and interpretation, and helped to draft the manuscript. All authors read and approved the final manuscript.

## Supplementary Material

Additional file 1: Table S1 Detailed medication exposures of study patients. Click here for file
